# Supplementary material for: Peli1 negatively regulates noncanonical NF-κB signaling to restrain systemic lupus erythematosus
Source: Nat Commun. 2018 Mar 19;9:1136. doi: 10.1038/s41467-018-03530-3 (PMC5859150; doi:10.1038/s41467-018-03530-3)
Supplement: Supplementary file 2 — Supplementary Information(PDF 2681 kb) [file 41467_2018_3530_MOESM2_ESM.pdf]

Supplementary Information

Peli1 negatively regulate noncanonical NF-κB signalling to restrain systemic lupus erythematosus

by Liu et al.

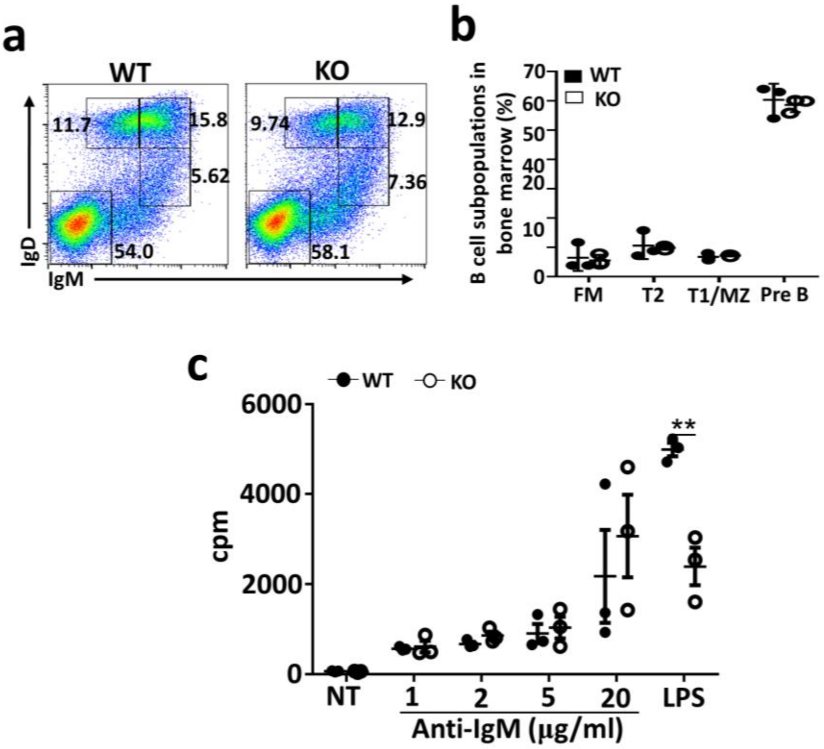

Supplementary Figure 1. Peli1 deficiency neither affect B cell development in bone marrow nor influence BCR-induced but impairs LPS-induced B cell proliferation. (**a**,**b**) Flow cytometric analysis of the percentages of B cell subpopulations, including $IgM^{low}IgD^{hi}$ follicular mantle (FM), $IgM^{hi}IgD^{hi}$ T2, $IgM^{hi}IgD^{med}$ T1/marginal zone (MZ), and $IgM^{hi}IgD^{hi}$ pre B cells, in bone marrows of WT and *Peli1*-dificient mice. Data are presented as a representative plot (**a**) and summary graph (**b**). (**c**) Proliferation of WT and KO splenic B cells incubated *in vitro* for 72 h in the absence (NT) or presence of different concentration of anti-IgM or LPS, then assessed by [3H]thymidine incorporation. Data are shown as the mean ± SEM based on three independent experiments. Two-tailed Student's t tests were performed. **P<0.01.

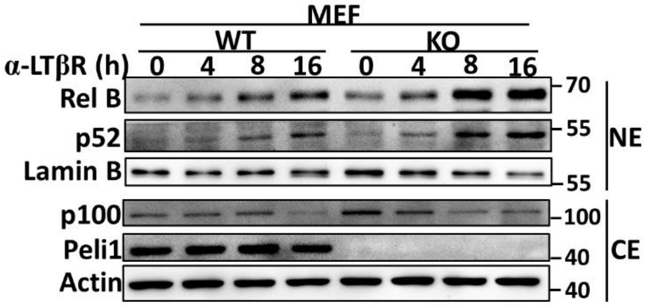

Supplementary Figure 2. Peli1 deficiency enhances anti-LTβR-induced noncanonical NF-κB activation in MEF. Immunoblot analysis of NF-κB proteins or Actin and lamin B (loading controls) in cytoplasmic extracts (CE) and nuclear extracts (NE) of MEF cells stimulated with anti-LTβR.

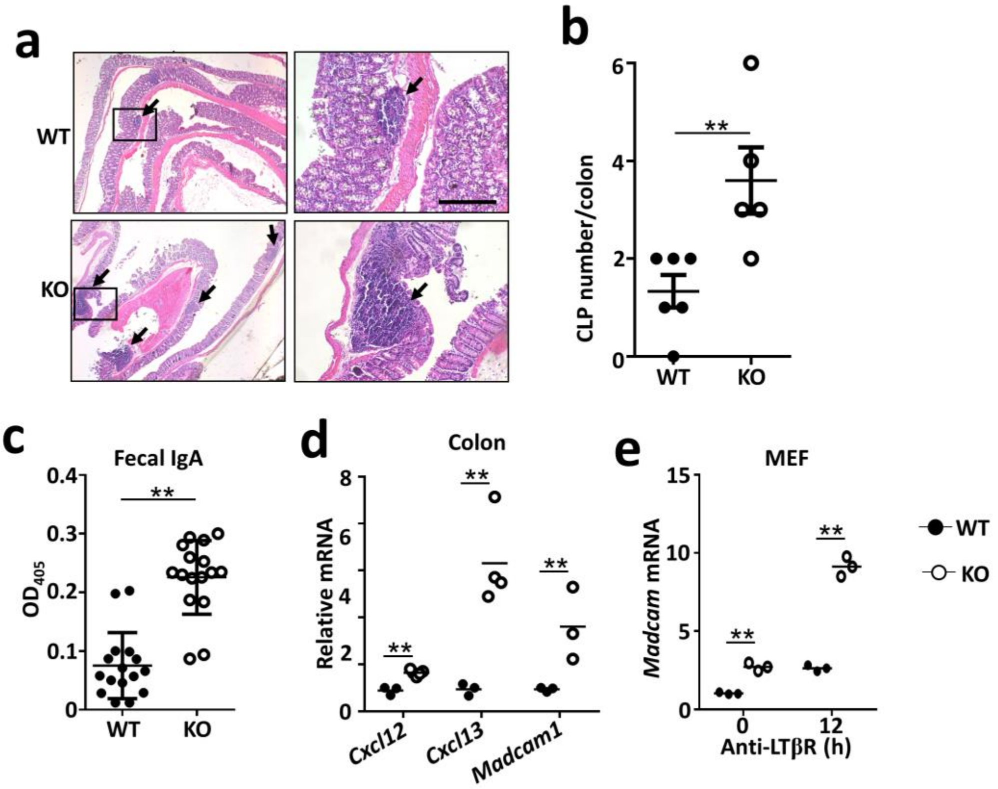

Supplementary Figure 3. Peli1 negatively regulates mucosal immunity. (**a,b**) Haematoxylin and eosin staining (**a**) and summary graph (**b**) of wild-type (WT) and Peli1-deficient (KO) colon. Black arrows point to CLPs. Scale bar, 100 μm. (**c**) ELISA of IgA in the faeces of WT and KO mice (n= 16). (**d,e**) QPCR assays using RNAs prepared from colonic tissues (**d**) and anti-LTβR-stimulated MEFs (**e**). Data are shown as the mean ± SEM based on three independent experiments. Two-tailed Student's t tests were performed. *P<0.05 and **P<0.01.

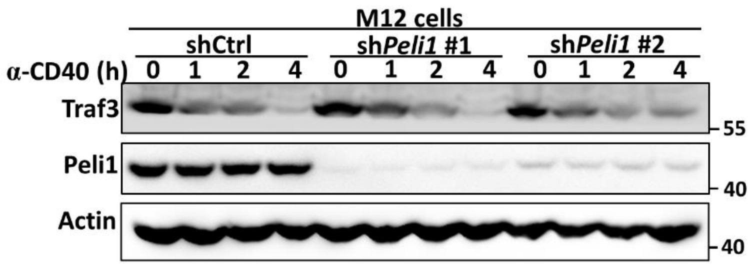

Supplementary Figure 4. Peli1 knockdown does not affect Traf3 degradation upon anti-CD40 stimulation in M12 cells. Immunoblot analysis of Traf3, Peli1 and Actin (loading controls) in whole cell lysis of M12 cells stimulated with anti-CD40.

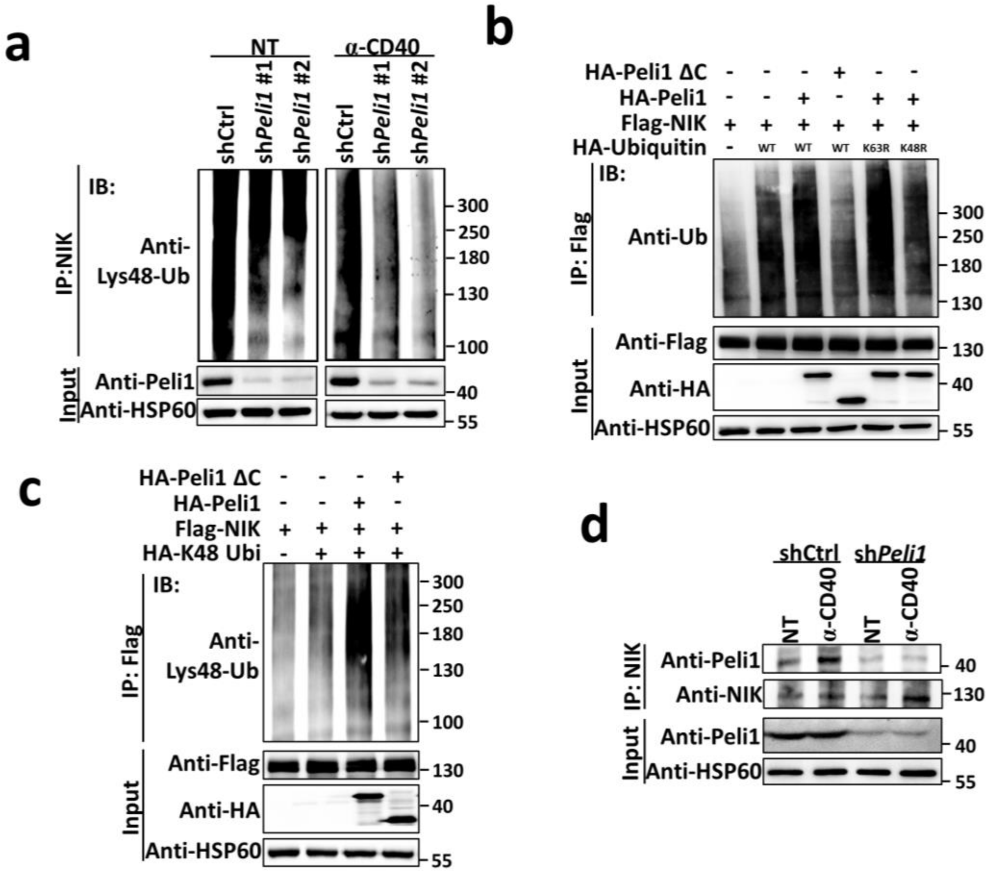

Supplementary Figure 5. Peli1 associates with NIK and mediates Lys48 ubiquitination of NIK. (**a**) Analysis of Lys48 ubiquitination of NIK in control and *Peli1*-knockdown M12 cells that left unstimulated (NT) stimulated with anti-CD40 (α-CD40) in the presence of a proteasome inhibitor MG132. IP, immunoprecipitation; IB, immunoblotting. (**b,c**) Analysis of the ubiquitination of NIK by using anti-ubiquitin or anti-Lys48 ubiquitin antibodies in HEK293 cells transfected with (+) or without (-) indicated expression vectors. (**d**) Immunoassays on lysates of WT and Peli1 knockdown M12 cells that left unstimulated or stimulated with anti-CD40 for 4 h in the presence of MG132, followed by IP with anti-NIK and immunoblot analysis of NIK-associated Peli1.

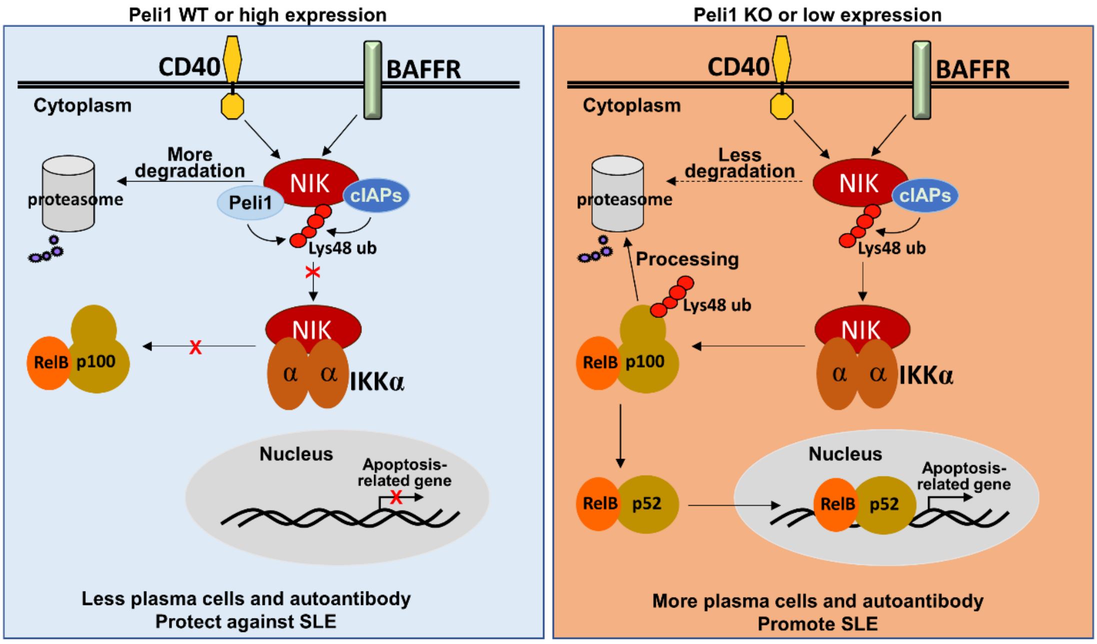

Supplementary Figure 6. Model depicting how Peli1 negatively regulate noncanonical NF-κB and protect against SLE. Upon activation from CD40 or BAFFR, Peli1, together with cIAPs, mediates Lys48 ubiquitination of NIK and promotes its degradation, leading to the inhibition of p100 processing to p52, and the suppressed nuclear translocation of RelB/p52 and noncanonical NF-κB activation. Thus, Peli1 WT or high expression inhibits the frequencies of plasma cells and autoantibody production through the suppression of noncanonical NF-κB signaling, resulting in the suppression of SLE pathogenesis. In contrast, Peli1 deficiency or low expression promoted the noncanonical NF-κB activation, and increased the frequencies of plasma cells and autoantibody production during SLE pathogenesis.

Fig2e

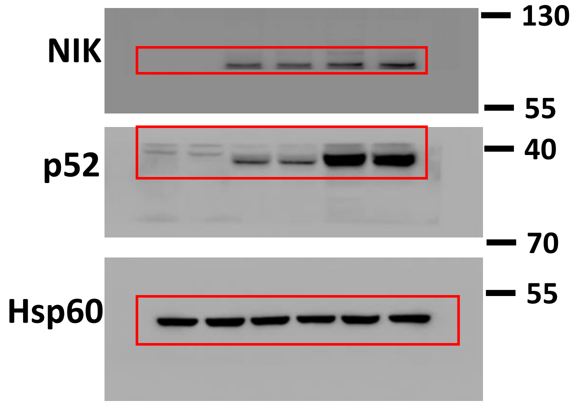

Fig4b

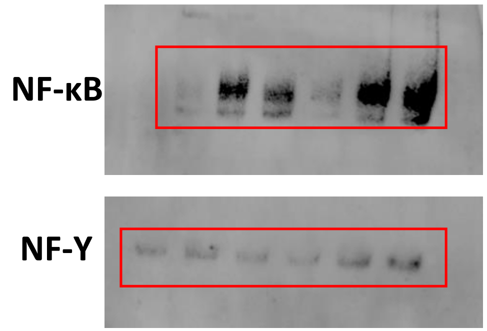

Fig4c

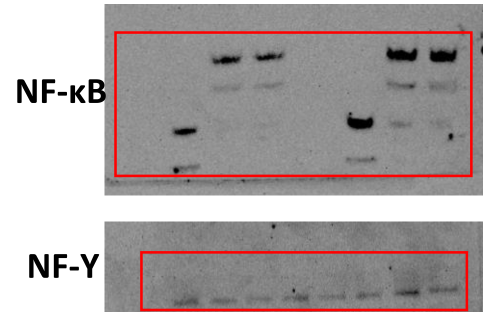

Fig4d

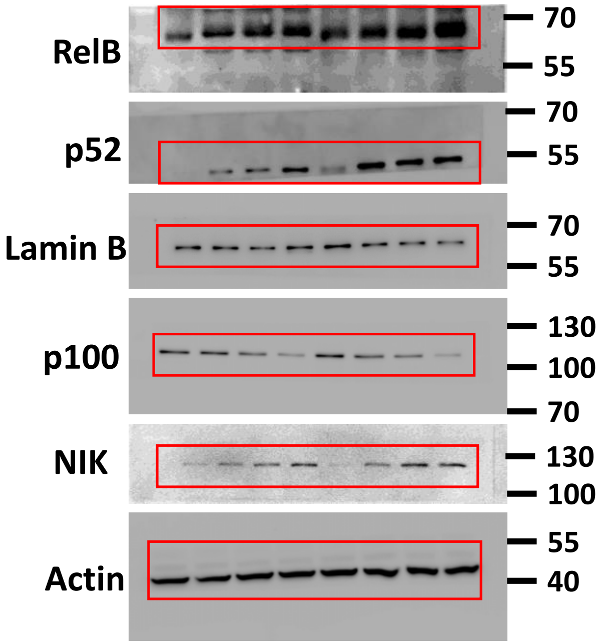

Fig4e

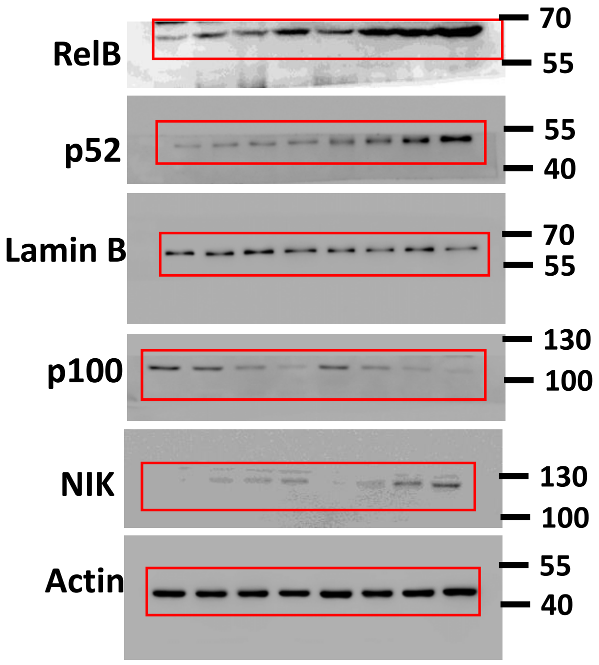

Fig4f

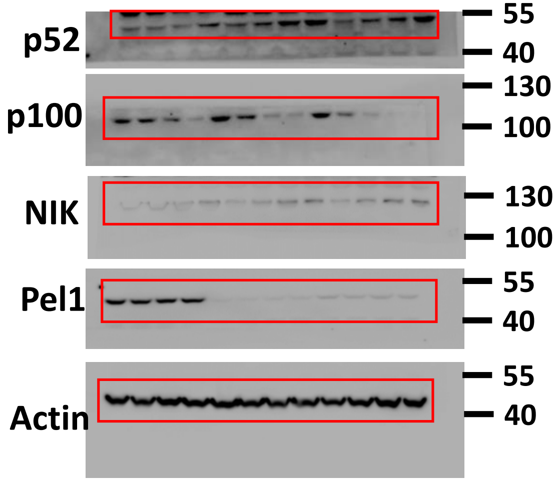

Fig4g

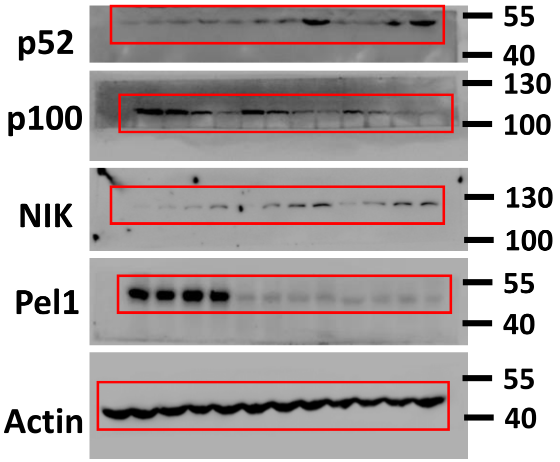

Fig4h

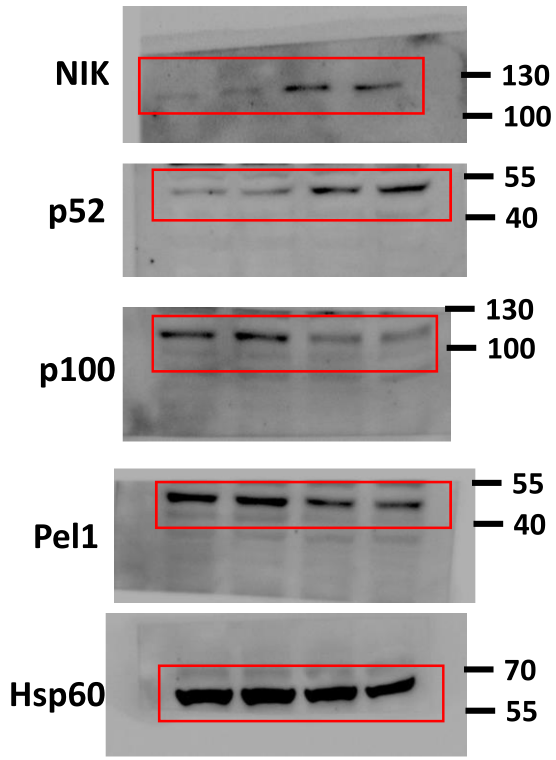

Fig4i

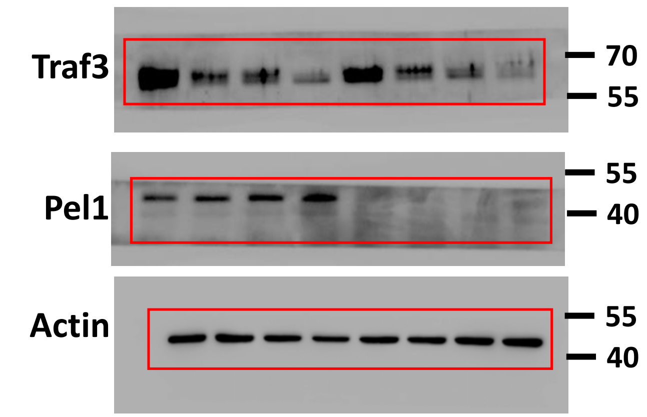

Fig6a

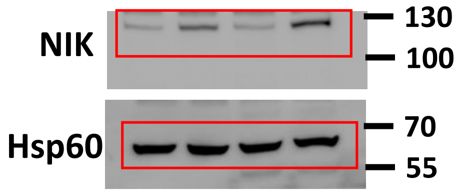

Fig6b

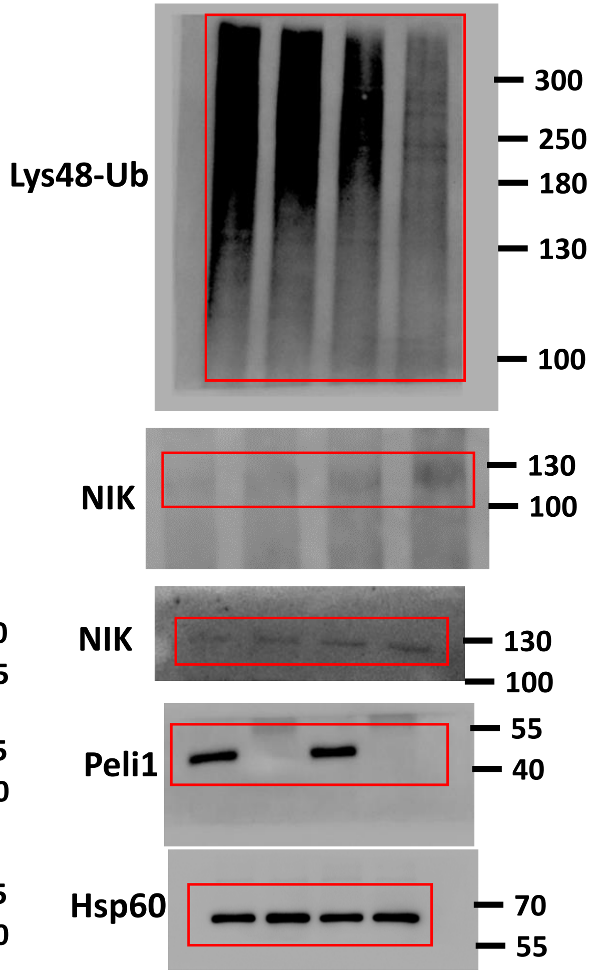

Fig6c

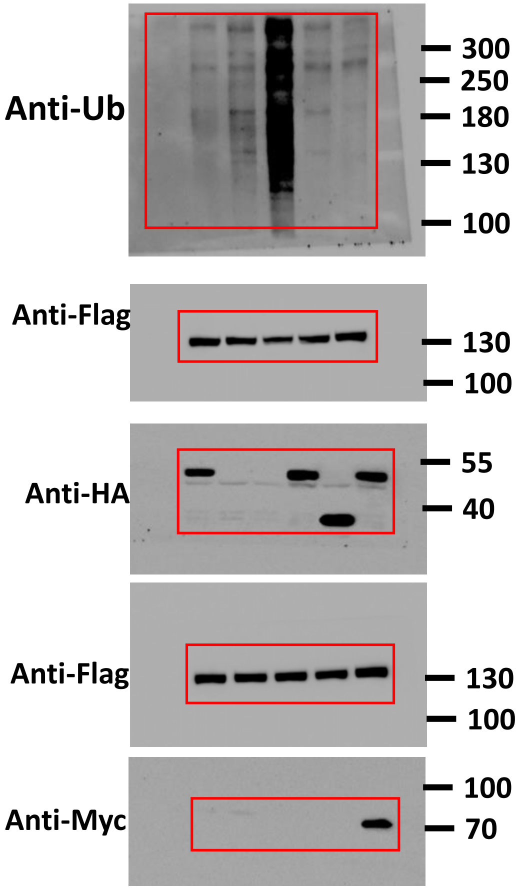

Fig6d

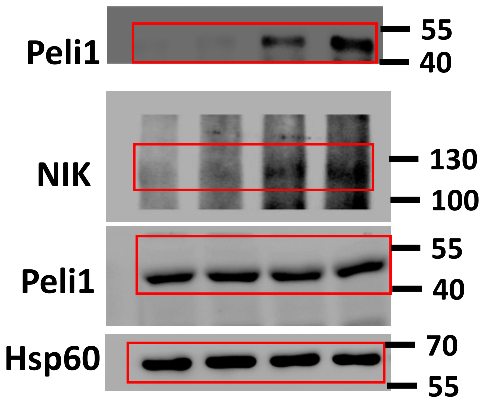

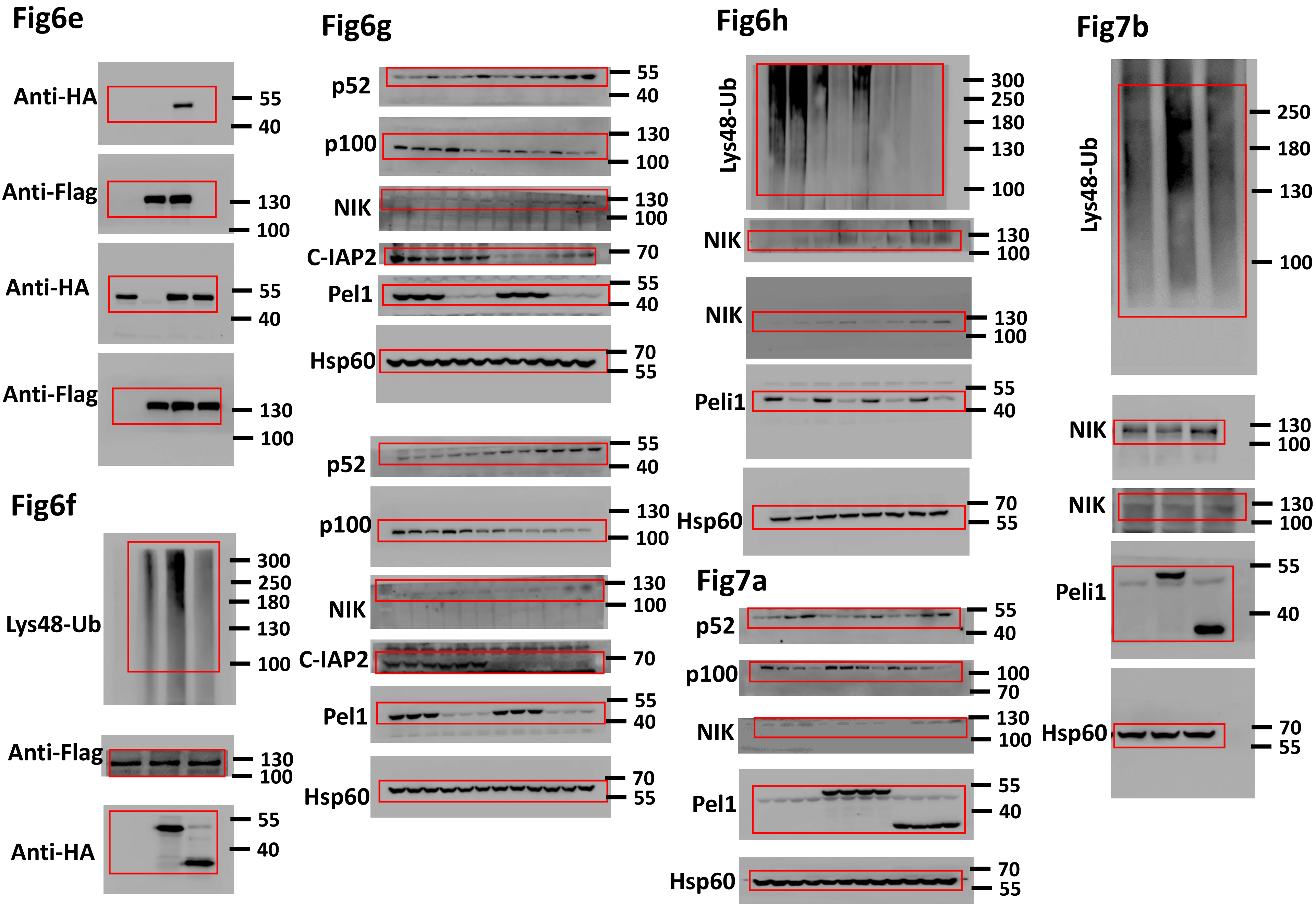

Supplementary Figure 7. All the uncropped scans of the western blots

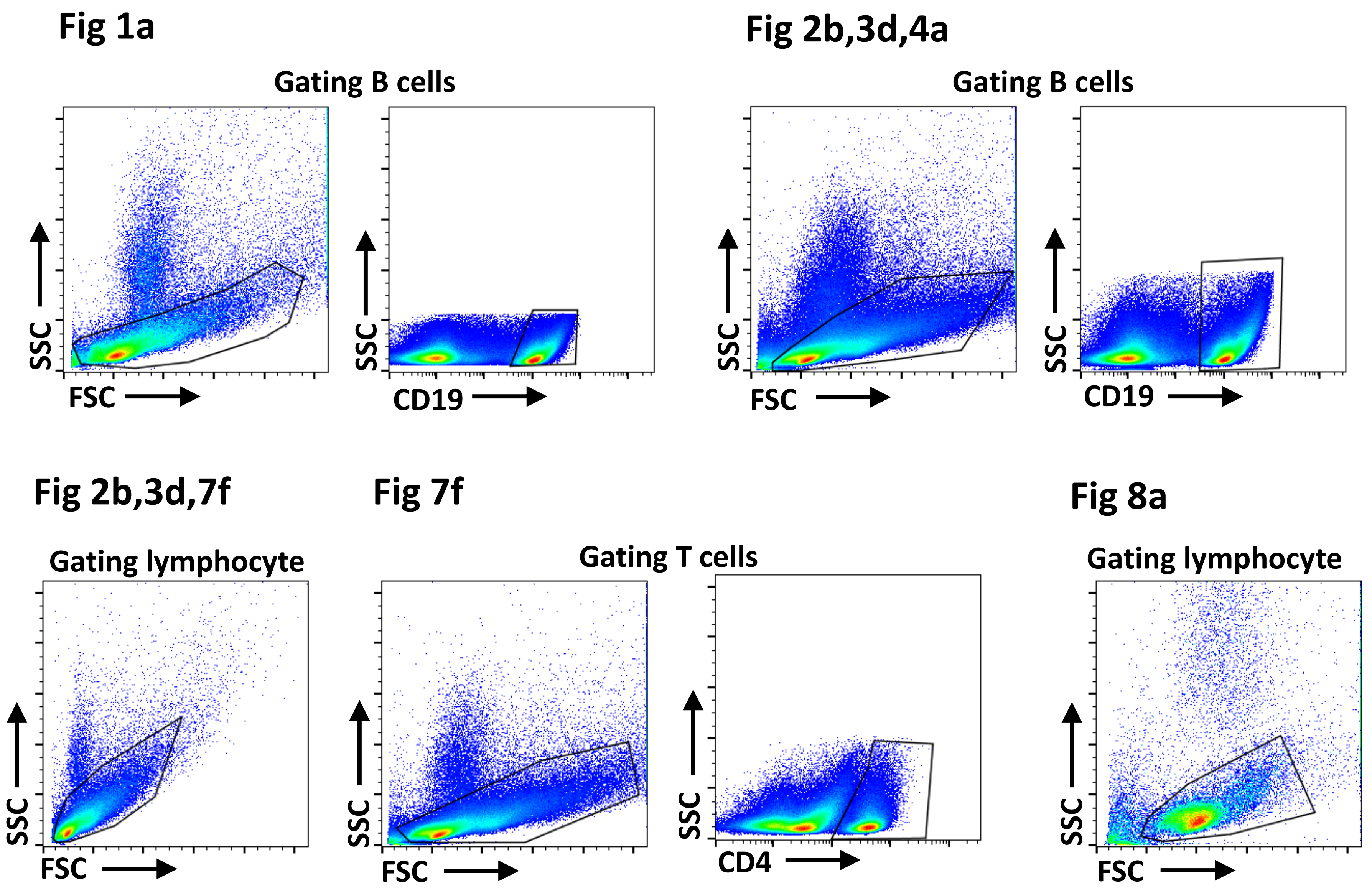

Supplementary Figure 8. All the FACS gating strategies

Supplementary Table 1. Primers used for real-time quantitative PCR

Genes	Forward primers	Reverse primers
h*Actin*	ACTCTTCCAGCCTTCCTTCC	CGTACAGGTCTTTGCGGATG
h*Peli1*	CGGCTCAGCAGAGAGGAAAA	TCACGGTAGGAGTGTGGGAA
h*Bclxl*	ACTCTTCCGGGATGGGGTAA	AGGTAAGTGGCCATCCAAGC
h*Bcl10*	AGTGAAGAAGGACGCCTTAGA	GGTGTCCAGACCTTTTGGGT
h*Cd22*	GCCATCCTCATCCTGGCAAT	GGGGGCCCTTCTAACCTTTT
h*Bcl11a*	CCAGCTCAAAAGAGGGCAGA	TCTGGGCACAGGCATAGTTG
h*Bcl7b*	TGCGGAAATGGGAGAAGAAGT	AAGCCATTAGGTTCTCGGGC
h*Bclaf1*	ACCGAAGGAAGAGGAATGGG	GAAGTTAAAGCGCCCTCTGC
h*Bcl2l12*	GAAGGAAGCCATACTGCGGA	CGAAAGAGTCGGAGGACAGG
m*Actin*	CGTGAAAAGATGACCCAGATCA	CACAGCCTGGATGGCTACGT
m*Cd22*	TCTCTCAGAAGGCCCCCAAT	AGTGTTGTGCGTGTCACTCT
m*Bclxl*	CCTTGGATCCAGGAGAACGG	CGACTGAAGAGTGAGCCCAG
m*Bcl11a*	GCGACACTTGTCCTTCACACA	TCTTGAGCTTCCATCCGAAAAC
m*Bcl2l12*	CCGCCTTGTGGAACTCTTCT	CCGGCTCAATTCCATGGCTA
m*Bcl2l15*	GCTAACCGGAACCTATCGGG	GTCCTCCAATGGTTACCGCA
m*Bclaf1*	GTCATCGTCCTCGTCAGCAT	CCAGCAAAAACTCCTCTGGC
m*Bcl10*	CTTCTCTATGGCGTCGTCCC	CCCTCTTCCAACCGAAGGTC
m*Cxcl12*	GCTCTGCATCAGTGACGGTA	TCAGATGCTTGACGTTGGCT
m*Cxcl13*	TTGGCACGAGGATTCACACA	ATTCAAGTTACGCCCCCTGG
m*Madcam1*	GAGCAAGAAGAGGAGATACAAGAG	TGGTGACCTGGCAGTGAAG

上海交通大学医学院附属新华医院医学伦理委员会

Ethics Committee of Xin Hua Hospital Affiliated to Shanghai Jiao Tong University School of Medicine

Approval No. XHEC-D-2018-012

The Ethical Review on the Research of 'Peli1 negatively regulate noncanonical NF-κB signalling to restrain systemic lupus erythematosus'

Dr. Xinfang Huang, et al. have submitted this research proposal entitled "**Peli1 negatively regulate noncanonical NF-κB signalling to restrain systemic lupus erythematosus**". In this study, by means of flow cytometry, immunoblot, RNA-sequencing and real-time quantitative PCR analysis, they examined the frequencies of plasma cells, the expression of non-canonical NF-κB related proteins, and RNA levels of related genes in the peripheral blood mononuclear cells (PBMCs) of systemic lupus erythematosus patients. This study may give some implications for the development of new therapeutic approaches. The research subjects in this study was part of the systemic lupus erythematosus patient cohort established on September 1st, 2016 by Renal and Rheumatology Division, Department of Internal Medicine, Xin Hua Hospital Affiliated to Shanghai Jiao Tong University School of Medicine and the last last follow-up of this study was August 31st, 2017. The PBMCs and biochemical data of the patients and health donors were collected after written informed consent were provided to all the recruited volunteers. The study protocol as well as the application form were fully reviewed and we have certified that this study did not raise any issues of patients' risk. We have also certified that the study was in accordance with the Declaration of Helsinki and conducted without ethics problems.

Ethics Committee of Xinhua Hospital Affiliated to Shanghai Jiaotong University School of Medicine

Feburary 5, 2018

上海交通大学医学院附属新华医院 医学伦理委员会

地址：上海市杨浦区控江路 1665 号 邮编： 电话：021-25076143 传真：021-25078922

Address:No.1665 Kongjiang Road Shanghai, P.R China,Postcode:200092 Tel: +86-21-25076143 Fax: +86-21-25078922
